# The Role and Potential Therapeutic Application of Myeloid-Derived Suppressor Cells in Allo- and Autoimmunity

**DOI:** 10.1155/2015/421927

**Published:** 2015-05-19

**Authors:** Qi Zhang, Masayuki Fujino, Jinhua Xu, Xiao-kang Li

**Affiliations:** ^1^Department of Dermatology, Huashan Hospital, Fudan University, Shanghai, China; ^2^Division of Transplantation Immunology, National Research Institute for Child Health and Development, Tokyo 157-8535, Japan; ^3^AIDS Research Center, National Institute of Infectious Diseases, Tokyo, Japan

## Abstract

Myeloid-derived suppressor cells (MDSCs) are a heterogeneous population of cells that consists of myeloid progenitor cells and immature myeloid cells. They have been identified as a cell population that may affect the activation of CD4^+^ and CD8^+^ T-cells to regulate the immune response negatively, which makes them attractive targets for the treatment of transplantation and autoimmune diseases. Several studies have suggested the potential suppressive effect of MDSCs on allo- and autoimmune responses. Conversely, MDSCs have also been found at various stages of differentiation, accumulating during pathological situations, not only during tumor development but also in a variety of inflammatory immune responses, bone marrow transplantation, and some autoimmune diseases. These findings appear to be contradictory. In this review, we summarize the roles of MDSCs in different transplantation and autoimmune diseases models as well as the potential to target these cells for therapeutic benefit.

## 1. Introduction

Suppressive myeloid cells were first described in the 1980s in patients with cancer [1–3]. With the subsequent research on this type of cells, a uniform name was suggested as myeloid-derived suppressor cells (MDSCs), reflecting their origin and function in 2007 [4]. MDSCs are a heterogeneous population of cells that consists of myeloid progenitor cells and immature myeloid cells [5]. They have the potential to affect the activation of CD4^+^ and CD8^+^ T-cells, leading to the negative regulation of the immune response, which makes them attractive targets for the treatment of transplantation and autoimmune diseases [6, 7]. Several studies have suggested the potential suppressive effect on alloimmune and autoimmune response [8, 9]. Conversely, MDSCs have also been found at various stages of differentiation, accumulating during pathological situations, not only during tumor development but also in a variety of inflammatory immune responses, bone marrow transplantation, and some autoimmune diseases [9].

These findings appear to be contradictory; are MDSCs beneficial or harmful for transplantation or autoimmune diseases and through what mechanisms? In this review, we summarize the roles of MDSCs in different transplantation and autoimmune diseases models as well as the potential to target these cells for therapeutic benefit.

## 2. Origin and Phenotype of MDSCs

Hematopoietic stem cells in the bone marrow give rise to myeloid precursor cells, and these cells generate “immature myeloid cells (IMCs)” without suppressive features. In healthy individuals, IMCs migrate into the peripheral lymphoid tissue, where they differentiate into mature macrophages, dendritic cells, or neutrophils [10]. In diverse pathologic processes, such as inflammation, tumors, infections, trauma, transplants, or autoimmune diseases, the differentiation of IMCs is inhibited. These cells are not abrogated to develop into functionally competent antigen presenting cells; instead, they are activated in response to tumors, pathogen-derived soluble factors, or host released cytokines [5, 11] and then differentiated into MDSCs, which produce immune suppressive factors such as arginase 1 (ARG1), inducible nitric oxidase synthase (iNOS), or reactive oxygen species (ROS) [5].

In mice, MDSCs are defined as CD11b^+^Gr1^+^ cells with suppressive functions and classified as either granulocytic MDSCs (G-MDSCs) (CD11b^+^Ly6G^+^Ly6C^low^) or monocytic MDSCs (M-MDSCs) (CD11b^+^Ly6G^−^Ly6C^hi^). The expression of the IL-4R *α*-chain (CD124), the monocytic marker CD115, low levels of the macrophage marker F4/80, and the stimulatory receptor CD40 have also been suggested as markers for MDSCs, although these markers are not unique and mostly lack relevance for identifying the suppressive population [12].

In humans, the criteria for identifying MDSCs in humans are still lacking, and phenotypic characterization of MDSC is even more difficult. The presence of MDSCs in cancer patients was first demonstrated nearly two decades ago [13]. Initial studies detected an increase in the number of myeloid origin cells in the peripheral blood of patients with squamous cell carcinomas of the head and neck (HNSCC) [14]. These cells were immature and expressed CD34 and could suppress the T-cell function [15]. Subsequent studies used different combinations of antigens including CD33, CD11b, HLA-DR, Lin, CD14, and CD15 to identify human MDSCs. While the expression of these markers has not been tested in all studies, most human MDSCs probably express both CD11b and CD33 and are negative for HLA-DR and Lin. Human MDSCs can also be divided into two groups: G-MDSCs and M-MDSCs. Human G-MDSCs generally express CD15, while M-MDSCs express CD14 [12].

## 3. The Role of MDSCs in Transplantation

### 3.1. Bone Marrow Transplantation

MDSCs are known to accumulate in lymphoid organs under conditions of intense immune activation. They also participate in the processes of bone marrow transplantation and graft-versus-host disease (GVHD). In the 1980s, the period in which MDSCs were named as “natural suppressor cells,” MDSCs were found to be increased in the spleen of bone marrow transplantation recipients and could significantly inhibit T-cell proliferation under the stimulation of alloantigens or mitogen* ex vivo* [16–19]. Billiau's group has elucidated much of the current knowledge of the relationship between MDSCs and BM chimeras. They found that the induction of BM chimerism in irradiated mice was associated with a transient expansion of CD11b^+^ Gr1^+^ cells with* in vitro* T-cell suppressive activity. The authors believed that the expansion most likely resulted from radiation-induced myelosuppression [20]. Billiau's group subsequently documented a similar expansion of CD11b^+^ Gr1^+^ myeloid progenitor cells in two parent-into-F1 models of chimerism induction [21]. These studies in mice showed that myeloid progenitor cells with suppressive capacity can expand as a physiological bystander phenomenon during the course of BM chimerism induction, suggesting a potential regulating role in the posttransplant immune environment. Furthermore, they also performed a detailed phenotypic and functional characterization of these cells in the two parent-into-F1 chimera models and found that the expanding CD11b^+^ myeloid progenitor cells comprise two phenotypically and functionally distinct MDSC subsets, CD11b^+^ Gr1^+^ Ly6C^+^ Ly6G^−^ cells and CD11b^+^ Gr1^+^ Ly6C^+^ Ly6G^+^ cells, and both MDSC subtypes were capable of regulating T-cell alloreactivity. This discovery nearly coincided with the aforementioned classification of M-MDSCs and G-MDSCs; they used the names of mononuclear (MO) MDSCs and polymorphonuclear (PMN) MDSCs to distinguish the two subsets and found suppressive effects of MO-MDSCs, but not PMN-MDSCs, involved in the production of iNOS [22].

In clinical allogeneic hematopoietic stem cell transplantation patients, Mougiakakos et al. showed that MDSCs can be found in allo-HSCT patients during the phase of immune reconstitution. They hypothesized that tissue damage following (radio)chemotherapy, as well as cytokines released from the cell transfer and subsequent immune (allo)reactions, creates a (cytokine-) milieu that favors the generation of MDSCs. They also characterized the CD14^+^HLA-DR^low/neg^ cells that accumulate in patients after allo-HSCT, especially during high-grade acute GHVD. The cell frequency significantly correlated with the serum levels of IL-6 and granulocyte-colony stimulating factor (G-CSF) and suppressed the proliferation of autologous T-cells in an indoleamine 2,3-dioxygenase- (IDO-) dependent manner [23].

G-CSF-mobilized peripheral blood mononuclear cells (G-PBMCs) have been widely used for autologous hematopoietic reconstitution after myeloablative therapy. G-CSF was also reported to be associated with MDSC induction. An early study by Mielcarek et al. found that when the donors were pretreated by G-CSF, G-CSF-mobilized blood cell grafts contained 50-fold more CD14^+^ cells and only 10-fold more T-cells than the marrow, and the increased CD14^+^ had an equivalent potency in suppressing the proliferative responses. They considered that the low incidence of GVHD after transplantation of allogeneic G-PBMCs was partially due to the mobilization of a large portion of immunosuppressive M-MDSCs [24]. Recently, another clinical study by Vendramin et al. reported the relevance of MDSCs in clinical acute GVHD. They found that systemic treatment with G-CSF induces an expansion of myeloid cells displaying the phenotype of M-MDSCs (Lin^low/neg^HLA-DR^−^CD11b^+^CD33^+^CD14^+^) with the ability to suppress alloreactive T-cells* in vitro*. Additionally, they evaluated whether the MDSC content in the peripheral blood stem cell grafts affected the occurrence of acute GVHD in patients undergoing unrelated donor allogeneic stem cell transplantation and found that the monocytic MDSC dose was the only graft parameter predictive of acute GVHD. Although further prospective studies involving larger sample sizes are needed to validate the optimal monocytic MDSC graft dose that protects from acute GVHD, their results strongly suggested that the modulation of G-CSF may be used to affect M-MDSCs graft cell doses to prevent acute GVHD [25].

Another study by Joo et al. showed that G-CSF induced CD11b^+^Gr1^+^ immune suppressive cells in mice, which inhibited acute GVHD lethality, but not through an IDO-dependent mechanism. These results suggested that there should be other mechanisms participating in the prevention of acute GVHD via the pretreatment of G-CSF induced MDSCs [26]. Following this speculation, a study by Highfill et al. showed that G-CSF and granulocyte-macrophage colony stimulating factor (GM-CSF), in conjunction with IL-13, could expand CD11b^+^Ly6G^lo^Ly6C^+^ MDSCs, and these cells suppressed GVHD dependent on L-arginine depletion by ARG1 activity. Exogenous IL-13 showed a strong supporting role, since the addition of exogenous IL-13 produced an MDSC subset that was more effective in preventing GVHD and demonstrated increased ARG1 activity [27].

In addition to G-CSF, other factors have been shown to influence the role of MDSCs during bone marrow transplantation. Morecki et al. showed that, in CpG-treated recipient mice, higher numbers of MDSCs were found, and these cells could reduce GVHD lethality compared with the control recipients [28]. Interestingly, extracorporeal photopheresis (ECP) was found to be beneficial for patients with GVHD; however, the underlying immunological mechanisms are not clearly understood. Rieber et al. found that ECP treatment in GVHD patients rapidly increased the circulating percentages of PMN-MDSCs. Functionally, PMN-MDSCs efficiently dampened the T helper (Th) type 1 (Th1) and Th17 responses, which was paralleled by an increase in cellular and extracellular arginase activity [29]. Conversely, Wang et al. investigated the relationship between MDSCs and GVHD development and demonstrated that the incidence of GVHD significantly enhances the number and suppressive function of MDSCs. Additionally, MDSC accumulation positively correlated with the severity of GVHD [30].

Moreover, one clinical investigation clarified that the administration of G-CSF, which is used to mobilize hematopoietic stem cells, induced an expansion of myeloid cells that displayed the phenotype of M-MDSCs (Lin^low/neg^HLA-DR^−^CD11b^**+**^CD33^**+**^CD14^**+**^) with the ability to suppress alloreactive T-cells* in vitro*. The monocytic MDSC dose was the only graft parameter predictive of acute GVHD. The modulation of G-CSF may thus be used to affect M-MDSCs graft cell doses for the prevention of acute GVHD [25].

However, MDSCs were found to be a double-edged sword for allogeneic BMT patients, as they negatively regulate GVHD development but also facilitate tumor growth [30]. Maintaining a delicate balance of MDSCs may present a challenging but promising approach for the control of GVHD and tumor relapse after allogeneic BMT.

### 3.2. Solid Organ Transplantation

In cardiac transplant models, several studies which potently achieved allograft tolerance by diverse treatments provided evidence that the increase in MDSCs contributed to the induction of indefinite allograft survival. Garcia et al. reported that when the recipients were treated with donor splenocyte transfusion (DST) in addition to anti-CD40L mAb to induce allograft tolerance, the bone marrow CD11b^+^CD115^+^Gr1^+^ M-MDSCs were mobilized and migrated from the bone marrow into the transplanted organs. They found that these MDSCs were necessary for tolerance induction. Additionally, MDSCs prevented the initiation of adaptive immune responses while inducing the development of regulatory T-cell (Tregs), which was dependent on IFN-*γ*R-iNOS signaling [31]. The conditioning regimen of total lymphoid irradiation (TLI) used with the T-cell depletive reagent, antithymocyte globulin/serum (ATG/ATS), has been shown to induce alloimmune tolerance in mice and humans after the development of persistent mixed chimerism. Hongo et al. discussed whether host MDSCs played an essential role in the development of chimerism and tolerance using TLI and ATS conditioning regimens in a murine cardiac transplantation model. The results of this study showed that the depletion of MDSCs abrogated chimerism and tolerance, and adding back these purified cells had a restorative effect, as MDSCs were required for the induction of chimerism and tolerance in the TLI and ATS regimens. Furthermore, MDSCs were activated to suppress alloreactivity by the direct or indirect interaction with host invariant (type I) NKT cells and IL-4 [32]. Ge et al. also used a murine cardiac transplant model and revealed that donor IL-6 deficiency significantly increased the infiltration of two MDSC subsets, CD11b^+^Gr1^−low^ and CD11b^+^Gr1^−int^, with strong immunosuppression activity in the transplanted graft, which resulted in a dramatic increase in the frequency of CD11b^+^Gr1^−low^ cells and a significant decrease of the frequency of CD11b^+^Gr1^−high^ and CD4^−^CD8^−^NK1.1^+^ cells in the recipient's spleen. This finding seems to conflict with other studies which showed that the phenotype of functional MDSCs was CD11b^+^Gr1^+^. In fact, the authors used an anti-Ly-6G mAb for the FACS staining of Gr1; therefore these results may suggest blocking of IL-6 induced regulatory G-MDSCs rather than M-MDSCs. However, their work did not determine the mechanism by which MDSCs play an immunosuppressive role in this model [33]. Recently, the same group tested the role of MDSCs in a murine presensitized skin and cardiac transplantation model. They revealed that the CD11b^+^Gr1^−low^ MDSCs subset, rather than the CD11b^+^ Gr1^−int^ or CD11b^+^ Gr1^−high^ subsets, showed immunosuppressive activity independent of Tregs; however, the mechanism by which the CD11b^+^ Gr1^−low^ MDSC subset regulated the alloimmune response was not determined [34]. Brunner et al. observed a significantly longer cardiac allograft survival in the recipients treated with IL-33, and a significant decrease in graft-infiltrating CD11b^high^Gr1^high^ granulocytes coincided with a significant increase in CD11b^high^Gr1^int^ MDSCs. In addition, this study showed that IL-33 treatment in the setting of chronic rejection promoted the development of a Th2-type immune response which may favor MDSCs and Tregs expansion, in addition to reduced antibody-mediated rejection (AMR) [35].

In kidney transplantation, Dugast et al. have demonstrated the accumulation of CD11b^+^CD80/86^+^ MDSCs in the peripheral blood in the anti-CD28 monoclonal antibody- (mAb-) induced rat kidney allograft tolerance model. These cells inhibited alloreactive T-cell proliferation and induced T-cell apoptosis in an iNOS-dependent manner. Although the adoptive transfer of these MDSCs isolated from the blood or the bone marrow did not significantly prolong the kidney allograft survival, the transfer still prevented the proliferation of allogeneic T-cells* in vivo* [36]. Subsequent work from the same group clarified the manner in which MDSCs cooperate with Tregs [37]. They compared the gene expression in blood-derived MDSCs from tolerant recipients of allogeneic kidney grafts using the same model with syngeneic grafts and observed the strong downregulation of CCL5 in blood MDSCs. Furthermore, they demonstrated the contribution of MDSCs to the establishment of a graft-to-periphery CCL5 gradient in tolerant kidney allograft recipients, which controls the recruitment of Tregs to the graft where they contribute to maintaining tolerance. In clinical studies, Hock et al. speculated the role of MDSCs in renal transplant recipients who have a high risk of cancer, particularly those with cutaneous squamous cell carcinoma. They demonstrated, for the first time, that renal transplant recipients and SCC patients had significantly elevated circulating levels of functional MDSCs and a systemic increase in the circulating MDSC/dendritic cell (DC) ratio. These results suggested the possibility that the increased MDSCs numbers may be a potentially useful marker to indicate renal transplant recipients with a higher level of functional immune suppression who are at an increased risk of cancer, but lower risk of transplant rejection [38]. Luan et al. demonstrated that CD11b^+^CD33^+^HLA-DR^−^ MDSCs are increased in renal transplanted patients while the* in vitro* immunosuppressive function is predominantly due to CD14^+^ M-MDSCs. More importantly, these MDSCs were capable of expanding Tregs* in vitro* and mediating the expansion of Foxp3-expressing Tregs in human kidney allograft recipients* in vivo* [39]. These findings were consistent with the results obtained from animal studies and clearly demonstrated the relationship between MDSCs and Tregs.

The regulatory role of MDSCs was also significantly demonstrated in skin transplant models. de Wilde et al. described that CD11b^+^GR-1^+^MDSC-compatible cells appeared after repetitive injections of lipopolysaccharide (LPS) in a skin transplantation model. These cells suppressed T-cell proliferation and Th1 and Th2 cytokine production in both mixed lymphocyte reaction and polyclonal stimulation assays. The transfer of CD11b^+^ cells from the LPS-treated mice in untreated recipients significantly prolonged the skin allograft survival. These cells produced excessive amounts of IL-10 and expressed heme oxygenase-1 (HO-1). HO-1 inhibition by the specific inhibitor tin protoporphyrin-IX (SnPP) completely abolished the T-cell suppression and IL-10 production. In contrast, neither iNOS nor ARG1 inhibition affected the suppression. This study was the first report to reveal the association between HO-1, a stress-responsive enzyme which possesses immunoregulatory and cytoprotective properties, and MDSC activity. Importantly, HO-1 inhibition before CD11b^+^ cell transfer prevented the delay of allograft rejection, thereby revealing a new MDSC-associated suppressor mechanism relevant to transplantation [40]. Zhang et al. found that the number and function of MDSCs were significantly enhanced by immunoglobulin-like transcript 2 (ILT2)* in vivo* during alloskin graft transplantation. They found that the interaction of human ILT2 receptor with its ligand* in vivo* created a microenvironment where immature myeloid cells develop; these ILT2-MDSCs expressed lower levels of MHC class I and higher levels of IL-4Ra. In addition, a histological evaluation of skin allografts showed that adoptively transferred MDSCs from ILT2 mice had a high capacity to migrate to the site of the graft, thus prolonging the allograft survival. These findings suggest that the exogenously activated immature myeloid cells may hold promise for human therapies [41]. Adeegbe et al. found that the administration of recombinant human G-CSF and interleukin-2 complex (IL-2C) induced Gr-1^+^CD11b^+^ MDSCs at a high frequency in the peripheral lymphoid compartments of treated mice. Interestingly, induced MDSCs exhibited a more potent suppressive function* in vitro* when compared to MDSCs from naive mice. The administration of G-CSF and IL-2C led to a significant delay of allogeneic donor skin rejection. Furthermore, the *ζ* chain expression by T-cells within the spleen of mice treated either with G-CSF or more markedly in combination with IL-2 was downregulated; thus the authors speculated that induced MDSCs may modify the T-cell phenotype via L-arginine metabolism-dependent mechanisms and/or disruption of the CD3 complex, which ultimately results in a lowered effector function [42]. Synthetic GC immunosuppressants, particularly dexamethasone, have been widely used in treating inflammatory disorders [43]. Liao et al. found that dexamethasone treatment upregulated the expression of chemokines that mediated CD11b^+^GR-1^+^ MDSCs recruitment, therefore prolonging the alloskin graft survival. These MDSCs suppressed the T-cell activation and modulated T-cell differentiation via NO production [44].

In addition to skin transplantation, MDSCs are observed in transplantation studies of other tissues. For instance, Chou et al. conducted cotransplantation with liver stromal cells (hepatic stellate cells (HSCs)) and achieved the long-term survival of islet allografts in mice via the induction of effector T-cell apoptosis and generation of Tregs. They analyzed the mechanism by which HSCs contribute to the prolongation of the allograft survival and found that HSCs could promote the generation of MDSCs* in vitro* and* in vivo*, which was dependent on an intact IFN-*γ* signaling pathway in HSCs [45].

Taken together, these studies suggest the role of MDSCs in the induction of several transplantation tolerance situations, not only by chemotherapeutic agents or biological antibodies, but also by presensitization or chimerism induction. MDSCs were also shown to be involved in the induction of alloimmune tolerance and in some cases played a pivotal role. Tregs are typically considered to be an essential factor in the induction of alloimmune tolerance, and the frequency of MDSCs was observed to correlate with the activity of Tregs; however, in some cases, they regulated the immune reaction by other mechanisms. These results indicate that the regulation of the immune microenvironment for maintaining immune homeostasis is the result of the induction of alloimmune tolerance. During the regulation process, MDSCs may have an independent immune regulation effect, similar to the role of immunoregulatory cells, such as Tregs. MDSCs may be either the reason or the result of the immune microenvironment regulating process, leading to a moderate interaction and circulation with other factors.

## 4. The Role of MDSCs in Autoimmunity

### 4.1. Experimental Autoimmune Encephalomyelitis

Experimental autoimmune encephalomyelitis (EAE) is a commonly used murine model of multiple sclerosis (MS). Using EAE models, several studies have examined the possible role of CD11b^+^Ly-6C^hi^ cells in this disease [46–48].

Different from the role of MDSCs in transplantation, the role of MDSCs in EAE revealed two completely opposite characteristics: (1) the accumulation of MDSCs positively correlated with the mouse EAE clinical score/disease severity, suggesting that CD11b^+^Ly6C^+^ cells may actually contribute to CNS damage, and (2) decreased pathology was associated with a reduction in the accumulation of CD11b^+^Ly-6C^hi^ monocytes in the CNS, suggesting that this cell population serves as pathologic effectors of the disease, rather than as suppressor cells [49]. King et al. found in a model of remitting/relapsing EAE that CD11b^+^ CD62L Ly-6C^hi^ cells accumulated in blood and trafficked across the blood-brain barrier into CNS prior to and during the course of EAE in myelin-immunized SJL mice [50]. The authors concluded that the enrichment of MDSCs was associated with an earlier onset and increased severity of clinical EAE, following their maturation into functional DCs and/or inflammatory macrophages. Yi et al. discovered that an excessive and prolonged presence of MDSCs can drive a Th17 response and consequently contributes to the pathogenesis of EAE [51]. MDSCs inhibited by gemcitabine result in a marked reduction in the severity of EAE (e.g., decreased clinical scores and myelin injury), which correlates with a reduction in the number of Th17 cells and inflammatory cytokines levels (IL-17A and IL-1b) in the lymphoid tissues and spinal cord. The adoptive transfer of MDSCs after gemcitabine treatment restores EAE disease progression. Mechanistic studies show that IL-1b represents a major mediator of MDSCs facilitated Th17 differentiation, which depends on the IL-1 receptor on CD4^+^ T-cells but not on MDSCs. These findings provide unique insights into the pleiotropic functions of MDSCs and may help explain the failure of immunosuppressive MDSCs to control Th17/IL-17-dependent autoimmune disorders. A study by Bruchard et al. also discussed the relationship of MDSCs and Th17 cells and showed MDSC-derived IL-1*β*-induced secretion of IL-17 by CD4^+^ T-cells, which diminished the anticancer efficacy of chemotherapy [52].

Conversely, Zhu et al. showed an opposite effect of MDSCs on EAE. They observed that splenic CD11b cells markedly increase after EAE immunization, and CD11b^+^Ly-6C^high^Ly-6G^−^ cells isolated from the spleen potently suppressed the proliferation of both CD4^+^ and CD8^+^ T-cells* in vitro* via the induction of T-cell apoptosis mediated by nitric oxide. These findings indicated that CD11b Ly-6C^high^ MDSCs induced during EAE priming are powerful suppressors of activated T-cells; however, this study examined* in vitro* effects of CD11b^+^Ly-6C^hi^ cells on T-cells alone and did not examine their effect* in vivo* (e.g., whether they participated in EAE pathology or suppressed the immune reaction) [53]. Furthermore, the presence and density of MDSCs and the proportion of apoptotic cells correlated with the EAE time course. The peak of the density paralleled the clinical score, decreased significantly during the remitting phase, and completely disappeared during the chronic phase. Furthermore, spinal cord-isolated MDSCs of EAE animals augmented the cell death when cocultured with stimulated control splenic T-cells [54]. In addition, two-week-old mice were resistant to active EAE, which causes fulminant paralysis in adult mice. Young resistant mice had higher frequencies of MDSCs and this resistance was associated with an impaired development of Th1 and Th17 cells [55]. Interestingly, these findings appeared to contradict the results of previous studies that showed that MDSCs could facilitate Th17 differentiation, which may be partially due to the underdeveloped immune systems of the young mice. However, the findings were consistent with the clinical observation that multiple sclerosis (MS) typically occurs in early adulthood while it is rare in children.

Moliné-Velázquez et al. demonstrated that MDSC polarization at a critical time of immunosuppression, induced by the differentiation agent Am80, affected the clinical course of EAE. Am80 induced MDSC apoptosis and caused a polarized MDSC cell phenotype, reflecting their maturation into myeloid cells and dampening their activity as immunosuppressors. These changes resulted in a substantial increase in the CD4^+^ T-cells (and probably other effector cells, i.e., macrophages and DCs) in the spleen and the spinal cord of EAE mice [56]. Taken together, these findings demonstrate that MDSCs are heterogeneous and plastic; therefore, specific cues in the microenvironment will preferentially activate specific subsets, functions, and pathways of differentiation.

### 4.2. Autoimmune Hepatitis

The liver appears to have an important role in MDSC biology. Sander et al. demonstrated that MDSCs have potent host-protective anti-inflammatory functions during polymicrobial infection and MDSCs functions during infection to hepatic acute-phase proteins (APPs) induced by gp130-STAT3 activation. They also showed that APPs are crucial regulators of the inflammatory responses to infection, highlighting the close relationship between hepatocytes and innate immune cells. Furthermore, serum amyloid A (SAA) plays a key role in this regulatory process. In conclusion, the present study adds important information on the role of the liver and hepatic APPs on the subsequent mobilization and accumulation of MDSCs, which in turn functions to inhibit pathologic inflammation [57].

Autoimmune hepatitis (AIH) is a liver-specific autoimmune disease in which T-cells express IFN-*γ* and accumulate in the liver portal tracts and parenchyma [58, 59], inducing hepatocellular damage and liver necrosis [60]. Cripps et al. demonstrated that liver inflammation mediated by Th1 cells can induce the accumulation of MDSCs. Isolated CD11b^+^Gr1^+^ myeloid cells from livers efficiently suppressed CD4^+^ T-cell proliferation* in vitro*. The suppressor function was dependent on cell-cell contact between MDSCs and T-cells, nitric oxide, and IFN-*γ*. The rapid accumulation of CD11b^+^Gr1^+^ cells in TGF-*β*1^−/−^ livers was abrogated when mice were either depleted of CD4^+^ T-cells or rendered unable to produce IFN-*γ*, demonstrating that Th1 activity induces MDSCs accumulation. These findings clarified that MDSCs serve an important negative feedback function in liver immune homeostasis and that insufficient or inappropriate activity of this cell population may contribute to inflammatory liver pathology [61].

Hammerich et al. showed that CREM*α* overexpression impaired the function of hepatic MDSCs and aggravated immune-mediated hepatitis in mice. Cremtg MDSCs isolated from the liver expressed reduced iNOS and ARG1 and displayed a reduced T-cell suppressive activity. The adoptive transfer of wild-type (wt) MDSCs was capable of reducing the fulminant immune-mediated liver damage in Cremtg mice to wt levels [62].

The presence of MDSCs in AIH patients has yet to be demonstrated. CD11b^+^ cells accumulation in the liver during AIH had been demonstrated by liver biopsy [63]. A recent study by Longhi et al. investigated 51 patients with autoimmune liver disease and 27 healthy subjects and found a higher number of monocytes with more vigorous spontaneous migration, which displayed higher TNF-*α* over IL-10 production [64].

### 4.3. Inflammatory Bowel Diseases

Inflammatory bowel diseases (IBD) include Crohn's disease and ulcerative colitis. Haile et al. previously described the development of a MDSC population in a murine model of IBD. This model of IBD was induced in transgenic mice harboring enterocyte-specific expression of hemagglutinin (HA) after the adoptive transfer of HA-specific CD8^+^ T-cells (CL4-TCR). The repeated transfer of HA-specific CD8^+^ T-cells prevented VILLIN-HA recipient mice from developing severe enterocolitis, which was seen after a single transfer of T-cells. Repeated transfer of antigen-specific T-cells led to an increase in the frequency of NOS2 and arginase-expressing CD11b^+^Gr-1^+^ MDSCs in the spleen and intestine of VILLIN-HA mice with immunosuppressive function. The cotransfer of MDSCs with HA-specific CD8^+^ T-cells into naive VILLIN-HA mice ameliorated enterocolitis, indicating a direct immune regulatory effect of MDSCs on the induction of IBD by antigen-specific T-cells. This cell population suppressed CD8^+^ T-cell proliferation* ex vivo* by the induction of T-cell apoptosis through a mechanism that required NO. Additionally, an increase in the frequency of human MDSCs with suppressor function was observed in the peripheral blood from patients with IBD. These results identify MDSCs as a new immune regulatory pathway in IBD [65, 66].

Guan et al. showed that the percentages of CD11b^+^Gr-1^+^MDSCs and other subsets (CD11b^+^Ly6C^+^ and CD11b^+^Ly6G^+^MDSCs) were increased in the spleen and/or colonic lamina propria mononuclear cells in colitis mice, which correlated with the severity of intestinal inflammation. However, MDSCs isolated from the colitis mice could suppress the proliferation of splenocytes* in vitro*. The adoptive transfer of MDSCs isolated from colitis mice decreased intestinal inflammation, the levels of IFN-*γ*, IL-17, and TNF, and the percentages of spleen MDSCs compared with the controls [67]. Thus, it appears that endogenous and exogenous MDSCs have different effects and may protect against inflammation or worsen inflammation depending on the context. This study also inspired the use of exogenous cultured MDSCs as a treatment for autoimmune diseases.

### 4.4. Other Autoimmune Diseases

Kurkó et al. reported an increased frequency of MDSC-like cells in the blood of patients with rheumatoid arthritis (RA) compared with healthy individuals and found a negative correlation between the frequencies of circulating MDSC-like and Th17 cells in RA patients [68]. This group also identified MDSCs with a predominant granulocytic phenotype in the synovial fluid (SF) of mice with proteoglycan-induced arthritis (PGIA, an autoimmune murine model of RA). In addition, they found that MDSCs were also present in the SF of RA patients. The majority of MDSCs in the SF of RA patients exhibited a neutrophil phenotype and morphology, similar to MDSCs identified earlier in the SF of mice with autoimmune arthritis. The suppression mediated by RA SF cells appears to be nonselective as these MDSCs potently suppress both the anti-CD3/CD28 Ab-induced and allo-Ag-induced proliferation of autologous blood T-cells [69].

McIntosh and Drachman described that a population of “large suppressive macrophages” (LSMs) was induced by restimulating spleen cells from rats with experimental autoimmune myasthenia gravis (EAMG)* in vitro* and the LSMs could induce apoptosis in activated T-cells [70]. Unfortunately, a phenotype analysis was not performed on these cells in order to distinguish whether or not these cells were MDSCs. As MDSCs were named for a cluster of cells which includes immune suppression cells, we speculate that the LSMs described in this study most likely include MDSCs.

MDSCs were also described in a murine model of experimental autoimmune uveoretinitis (EAU), an autoimmune intraocular inflammatory disease. They were found to resemble monocytes, expressed CD11b, and accumulated in conjunction with the progression of inflammation in the eye. The inflamed eye also contains a considerable proportion of Foxp3^+^ regulatory cells.* In vitro*, cells derived from the inflamed eye were shown to inhibit the proliferation of activated T-cells [71]. Subsequent studies from this group showed that the suppressive function of MDSCs in EAU required an intact TNF response axis [72].

CD11b^+^Gr1^low^ cells were identified in MRL-Fas^lpr^ mice, which resemble human systemic lupus erythematosus (SLE). These cells increased in the kidney and blood during disease progression and had a suppressive effect on CD4^+^ T-cell proliferation, which was restored by an ARG1 inhibitor. Arginase, rather than iNOS, mediated the suppression by MDSCs in this murine model [73]. Furthermore, Lourenço et al. reported that laquinimod administration in a (NZB × NZW) F1 murine model of SLE revealed the prevention or delay of lupus manifestations, which was associated with reduced numbers of monocyte/macrophages, dendritic cells, and lymphocytes, as well as the induction of MDSCs in the spleen and kidney. Furthermore, the production of IL-10 was induced and a decreased expression of TNF-*α*, IFN-*γ*, and IL-17 was observed [74].

Singh et al. have shown that MDSCs can be experimentally elicited in the context of a murine model of the autoimmune disease alopecia areata, a hair follicle-affecting autoimmune disease, in which the inflammatory immune pathology leads to hair loss. These Gr1^+^CD11b^+^ cells were able to inhibit T-cell proliferation* in vitro* and subsequent* in vivo* application could lead to partial restoration of hair growth [75].

Whitfield-Larry et al. showed that MDSCs were increased in frequency, but not maximally suppressive, in the peripheral blood of type 1 diabetes mellitus patients [76]. Gao et al. hypothesized that MDSCs played a role in the resistance to diabetes in the absence of complement C3. Indeed, the number of MDSCs was significantly increased in streptozotocin- (STZ-) treated C32/2 mice. These cells highly expressed ARG1 and iNOS. Importantly, the depletion of MDSCs led to the occurrence of overt diabetes in C32/2 mice after STZ treatment. Furthermore, C32/2 MDSCs actively suppressed diabetogenic T-cell proliferation and prevented/delayed the development of diabetes in an arginase and/or iNOS-dependent manner. Both Tregs and TGF-*β* were crucial for MDSCs induction in STZ-treated C32/2 mice [77].

## 5. Therapeutic Potential of Induced MDSCs in Transplantation and Autoimmunity

Through numerous scientific research experiences, MDSCs were found to have potential therapeutic effects on allo- and autoimmune reactions; however, endogenous and exogenous MDSCs appeared to play different roles in many situations; in some autoimmune diseases, endogenous MDSCs aggravated the diseases whereas exogenous MDSCs suppressed the immune response to alleviate the conditions. Therefore, the* in vitro* induction of functionally suppressive MDSCs would be important for utilizing MDSCs. Several groups have shown the induction of MDSCs originating from various cell types using different induction methods.

Qian et al. demonstrated that HSCs could promote the generation of MDSCs both* in vitro* and* in vivo*. Cotransplantation with HSCs achieved the long-term survival of islet allografts in mice through the induction of effector T-cell apoptosis and generation of Tregs. The induction of MDSCs is dependent on an intact IFN-*γ* signaling pathway in HSCs and is mediated by soluble factors, suggesting that specific tissue stromal cells, such as HSCs, play a crucial role in regulating the immune response via inflammation-induced generation of MDSCs [45].

The authors also conducted diverse experiments to investigate the potential therapeutic application of HSC-generated MDSCs from bone marrow cells and the underlying mechanisms. In one study, bone marrow cells were isolated from wt or iNOS^−/−^ mice and cultured in the presence of GM-CSF and HSCs, resulting in the generation of MDSCs. Wt or iNOS^−/−^ MDSCs were cotransplanted with islet allografts under the renal capsule of diabetic recipient mice. The addition of HSCs in DC cultures promoted the generation of MDSCs instead of DCs. MDSCs had elevated expression levels of iNOS upon exposure to IFN-*γ* and inhibited T-cell responses. Cotransplantation with wt MDSCs markedly prolonged the survival of islet allografts, which was associated with reduced infiltration of CD8^+^ T-cells resulting from an inhibited proliferative response. These effects were significantly attenuated when MDSCs had deficient iNOS expression levels. Furthermore, iNOS^−/−^ MDSCs largely lost their ability to protect islet allografts [78].

In another study, HSC-generated MDSCs were also mixed with islet allografts and transplanted into diabetic recipients. This study showed that cotransplantation with MDSCs, but not DCs, effectively protected the islet allografts without the requirement of immunosuppression, which was associated with the attenuation of CD8^+^ T-cells in the grafts and marked expansion of Tregs. Both* in vitro* and* in vivo* data demonstrated that B7-H1 (PD-L1) was absolutely required for MDSCs to exert immune regulatory activity and the induction of Treg cells [79].

Additional studies by this group also found that the adoptive transfer of these MDSCs effectively reversed the disease progression in experimental autoimmune myasthenia gravis (EAMG), a T-cell-dependent and B-cell-mediated model of myasthenia gravis. In addition to an ameliorated disease severity, MDSC-treated EAMG mice showed suppressed acetylcholine receptor- (AChR-) specific T-cell responses, decreased levels of serum anti-AChR IgGs, and reduced complement activation at the neuromuscular junctions.

MDSCs directly inhibit B-cells through multiple mechanisms, including PGE2, iNOS, and arginase and inhibit AChR-specific immune responses at least partially in an Ag-specific manner [80].

In addition, besides HSCs from the hepatic environment, the authors attempted to investigate other stromal cells from diverse tissues. For instance, they found that retinal pigment epithelial cells (RPEs) inhibited DC propagation and induced MDSCs differentiation from myeloid progenitor cells in bone marrow (BM) cells. The RPE-induced MDSCs were CD11b^+^Gr-1^+^ and had profound T-cell inhibitory activities. The lack of B7-H1 (PD-L1) on RPEs did not alter the numbers of RPE-induced MDSCs, nor did blocking the activities of TGF-*β* or CTLA-2*α*. However, blocking IL-6 in the RPE-BM cell cocultures significantly inhibited MDSC differentiation, suggesting that IL-6 is important for RPEs to induce MDSCs. Additionally, the adoptive transfer of RPE-induced MDSCs significantly inhibited autoreactive T-cell responses that lead to retinal injury in EAU [81].

We have previously illustrated a feasible approach for generating functional regulatory DCs from murine iPS cells [82]. Future experiments will focus on generating MDSCs from iPS cells in an attempt to apply the iPS-MDSCs to solid organ transplantations, such as the heart, liver, and kidney. iPS cells are very similar to embryonic stem (ES) cells in many respects, including gene expression patterns and pluripotent characteristics; however, they are not restricted by the same ethical concerns as ES cells. Therefore, iPS cells have great potential as a major cell source for the production of various types of cells or organs in regenerative medicine [83, 84].

The* in vitro* generation of MDSCs has also been conducted by other groups using innovative ideas and methods.

Kurkó et al. demonstrated that BM cells cultured in the presence of GM-CSF, IL-6, and G-CSF became enriched in MDSC-like cells that showed greater phenotypic heterogeneity than MDSCs present in the SF. BM-MDSCs profoundly inhibited both antigen-specific and polyclonal T-cell proliferation primarily via the production of nitric oxide. The injection of BM-MDSCs into mice with PGIA ameliorated arthritis and reduced PG-specific T-cell responses and serum antibody levels [69].

In the study by Su et al., which detected the role of MDSCs in a TNBS-induced colitis model, the adoptive transfer of GM-CSF-induced MDSCs from BM cells* in vitro* ameliorated TNBS-induced intestinal inflammation and downregulated the levels of proinflammatory cytokines of recipient mice with colitis [85].

Mesenchymal stromal cells (MSCs) are multilineage progenitors with immunomodulatory properties, including the expansion of immunomodulatory leukocytes such as Tregs and tolerogenic DCs [86]. Yen et al. reported that human MSCs can expand CD14^−^CD11b^+^CD33^+^ human MDSCs. MSC-expanded MDSCs suppressed allogeneic lymphocyte proliferation, expressed ARG-1 and iNOS, and increased the number of Tregs. This expansion occurred through the secretion of hepatocyte growth factor (HGF); similar effects were replicated by the addition of HGF and abrogated by HGF knockdown in MSCs. The expansion of MDSCs by MSC-secreted HGF involves c-Met (its receptor) and downstream phosphorylation of STAT3, a key factor in MDSC expansion [87]. These data further support the strong immunomodulatory nature of MSCs and demonstrate the role of HGF, a mitogenic molecule, in the expansion of MDSCs.

Zoso et al. described and characterized fibrocystic MDSCs, which are differentiated from umbilical cord blood precursors by culture with human-GM-CSF and human-G-CSF. This MDSC subset is characterized by the expression of MDSC-, DC-, and fibrocyte-associated markers, promoted Treg-cell expansion, and induced normoglycemia in a xenogeneic murine model of type 1 diabetes [88].

Zhou et al. showed that functional MDSCs can be efficiently generated from mouse embryonic stem (ES) cells and BM hematopoietic stem (HS) cells.* In vitro* derived MDSCs encompass two homogenous subpopulations: CD115^+^Ly-6C^+^ and CD115^+^Ly-6C^−^ cells. The CD115^+^Ly-6C^+^ subset is equivalent to the monocytic Gr-1^+^CD115^+^F4/80^+^ MDSCs found in tumor-bearing mice. In contrast, the CD115^+^Ly-6C^−^ subset, a previously unreported population of MDSCs, developmentally resembles granulocyte/macrophage progenitors.* In vitro*, ES- and HS-MDSCs exhibit robust suppression against T-cell proliferation induced by polyclonal stimuli or alloantigens via multiple mechanisms involving nitric oxide synthase-mediated NO production and IL-10. Impressively, these cells displayed even stronger suppressive activity and significantly enhanced ability to induce CD4^+^CD25^+^Foxp3^+^ Treg development compared with tumor-derived MDSCs. Furthermore, the adoptive transfer of ES-MDSCs could effectively prevent alloreactive T-cell-mediated lethal GVHD, leading to nearly 82% long-term survival among treated mice [89].

Moreover, Obermajer and Kalinski described a simple and clinically compatible method of generating large numbers of MDSCs using the cultures of PBMCs supplemented with prostaglandin E2 (PGE2). They observed that PGE2 induces endogenous cyclooxygenase-2 (COX-2) expression in cultured monocytes, blocking their differentiation into CD1a^+^ DCs and inducing the expression of indoleamine 2,3-dioxygenase 1, IL-4R*α*, nitric oxide synthase 2, and IL-10, typical MDSC-associated suppressive factors. The establishment of a positive feedback loop between PGE2 and COX-2, the key regulator of PGE2 synthesis, is both necessary and sufficient to promote the development of CD1a^+^ DCs to CD14^+^CD33^+^CD34^+^ M-MDSCs in GM-CSF/IL-4-supplemented monocyte cultures. In addition to PGE2, selective E-prostanoid receptor (EP)2- and EP4-agonists, but not EP3/1 agonists, also induce the development of MDSCs, suggesting that other activators of the EP2/4- and EP2/4-driven signaling pathway (e.g., adenylate cyclase/cAMP/PKA/CREB) may be used to promote the development of suppressive cells [90].

## 6. Concluding Remarks

MDSCs are a highly heterogeneous cell subpopulation. The expansion and activation of MDSCs* in vivo* are dependent on which models are utilized and the local microenvironments [53]. The immunosuppressive effects of MDSCs have been shown through various pathways such as iNOS, ARG1, IDO, HO-1, and IL-10. MDSCs are certainly involved in the induction of transplantation immune tolerance, which makes the use of MDSCs an attractive therapeutic application for controlling graft rejection and establishing the induction of transplantation tolerance (Table 1). However, the presence of MDSCs in autoimmune diseases is different, and current studies showed conflicting roles for MDSCs in autoimmunity, either as an aggravating or as a curative factor of disease (Tables 1 and 2). Specifically, endogenous MDSCs showed acceleration, rather than deceleration, of immunoreaction, whereas exogenous MDSCs showed effective suppression. One hypothesis is that the dysfunction of MDSCs* in vivo* leads to the accumulation of MDSCs in response to inflammation; however, MDSCs fail to effectively downregulate the T-cell response, resulting in additional inflammation and additional dysfunctional MDSCs. Some factors within the inflammatory microenvironment may prevent pre-MDSCs from realizing their suppressive potential and the removal of MDSCs from this “inhibitory” environment allows the suppressor phenotype to emerge, rendering them functional upon readministration (and presumably resistant to further inhibition). Further investigations on the molecular mechanisms for the biological properties of MDSCs are necessary.

## Figures and Tables

**Table 1:** Suppressive MDSCs in different transplantation and autoimmune disease models.

	Species	Diseases or models	Cell surface phenotype	Inducing factors	Mechanism of suppression	Reference
1	Mouse	GVHD	CD11b^+^Gr-1^+^	Radiation-induced BM chimerism	Unknown	[20]

2	Mouse	GVHD	CD11b^+^Gr-1^+^	Two parent-in-F1 models of chimerism	Inducible nitric oxidase synthase (iNOS)	[21]

3	Human	Allo-HSCT patient	CD14^+^HLA-DR^low/neg^	Radiochemotherapy	Indoleamine 2,3-dioxygenase (IDO)	[23]

4	Human	GVHD	Lin^low/neg^HLA-DR^−^CD11b^+^CD33^+^CD14^+^	Granulocyte-colony stimulating factor (G-CSF)	Dose dependent	[25]

5	Mouse	GVHD	CD11b^+^Gr-1^+^	G-CSF	Unknown but not IDO	[26]

6	Mouse	GVHD	CD11b^+^Ly6G^lo^Ly6C^+^	G-CSF and granulocyte-macrophage colony stimulating factor (GM-CSF) with interleukin- (IL-) 13	Arginase-1 (Arg-1)	[27]

7	Mouse	GVHD	CD11b^+^Gr-1^+^	CpG	Unknown	[28]

8	Human	GVHD	HLA-DR^low^CD124^+^/IL-4R*α* ^+^ CD184^+^/CXCR4^+^	ECP	Arg-1	[29]

9	Mouse	Heart transplantation	CD11b^+^CD115^+^Gr1^+^	Donor splenocyte transfusion (DST) plus anti-CD40L mAb	Tregs development, dependent on IFN-*γ*R-iNOS signaling	[31]

10	Mouse	Heart transplantation	CD11b^+^Gr-1^+^	Total lymphoid irradiation (TLI) and antithymocyte globulin/serum (ATG/ATS)	Tregs, NKT, arginase-1, IL-4R*α*, and PDL1	[32]

11	Mouse	Heart transplantation	CD11b^+^Gr1^−low^ and CD11b^+^Gr1^−int^	Donor IL-6 deficiency	Unknown	[33]

12	Mouse	Heart transplantation	CD11b^+^Gr1^−low^	Presensitized skin transplantation	Unknown but independent of Tregs	[34]

13	Mouse	Heart transplantation	CD11b^high^Gr1^int^	IL-33	Tregs	[35]

14	Rat	Kidney transplantation	CD11b^+^CD80/86^+^	Anti-CD28	iNOS-dependent	[36]

15	Rat	Kidney transplantation	CD11b^+^CD80/86^+^	Anti-CD28	Tregs, CCL-5	[37, 38]

16	Human	Kidney transplantation	CD11b^+^CD33^+^HLA-DR^−^	Tacrolimus/MMF, prednisone	Tregs	[39]

17	Mouse	Skin transplantation	CD11b^+^GR-1^+^	LPS	IL-10, HO-1	[40]

18	Mouse	Skin transplantation	CD11b^+^GR-1^+^	Immunoglobulin-like transcript 2 (ILT2)	MHC class I, IL-4Ra	[41]

19	Mouse	Skin transplantation	CD11b^+^GR-1^+^	Human G-CSF, interleukin-2 complex (IL-2C)	T-cell *ζ* chain downregulation	[42]

20	Mouse	Skin transplantation	CD11b^+^GR-1^+^	Dexamethasone	Nitric oxide (NO)	[44]

21	Mouse	Islet allograft	CD11b^+^CD11c^+^	Hepatic stellate cells (HSC)	Treg IFN-*γ* signaling	[45]

22	Mouse	Experimental autoimmune encephalomyelitis (EAE)	CD11b^+^Ly-6C^high^Ly-6G^−^	/	T cell apoptosis, NO	[53]

23	Mouse	Autoimmune hepatitis	CD11b^+^GR-1^+^	Hepatic acute-phase proteins (APPs)	gp130–STAT3 activation	[57]

24	Mouse	Autoimmune hepatitis	CD11b^+^GR-1^+^	Th1 cells	NO, IFN-*γ*	[61]

25	Mouse	Inflammatory bowel diseases (IBD)	CD11b^+^GR-1^+^	Adoptive transfer of hemagglutinin- (HA-) specific CD8^+^ T cells	T cell apoptosis, NO	[65, 66]

26	Mouse	Experimental autoimmune myasthenia gravis (EAMG)	Unknown	/	T cell apoptosis	[70]

27	Mouse	Experimental autoimmune uveoretinitis (EAU)	CD11b^+^	/	Tregs, TNF response axis	[71, 72]

28	MRL-Fas^lpr^ mice	Systemic lupus erythematosus (SLE)	CD11b^+^Gr1^low^	/	Arg-1	[73]

29	(NZB × NZW) F1 mouse	SLE	CD11b^+^Gr1^+^	Laquinimod	Unknown	[74]

30	Mouse	Autoimmune disease alopecia areata	CD11b^+^Gr1^+^	/	T cell apoptosis	[75]

31	C32/2 mice	Diabetes	CD11b^+^Gr1^+^	Streptozotocin (STZ)	Arg-1, iNOS, Tregs, and transforming growth factor-*β* (TGF-*β*)	[77]

32	Mouse	Islet transplantation	CD11b^+^Gr1^+^	Hepatic stellate cells (HSC)	Tregs, IFN*γ* signaling pathway	[45]

33	Mouse	Islet transplantation	CD11b^+^Gr1^+^	HSC, GM-CSF	iNOS, IFN*γ* signaling pathway	[78]

34	Mouse	EAMG	CD11b^+^Gr1^+^	Acetylcholine	PGE2, iNOs, Arg-1,	[80]

35	Mouse	EAU	CD11b^+^Gr1^+^	Retinal pigment epithelial (RPE) cells	IL-6	[81]

36	Mouse	Arthritis	CD11b^+^Gr1^+^	M-CSF, IL-6, and G-CSF	NO	[69]

37	Mouse	TNBS-induced colitis model	CD11b^+^Ly6G^+^Ly6C^low^	GM-CSF	Unknown	[85]

38	Human	/	CD11b^+^Ly6G^+^Ly6C^low^	Mesenchymal stromal cells (MSC)	ARG-1, iNOs, hepatocyte growth factor (HGF)	[87]

39	Mouse	Xenogeneic model of type 1 diabetes	CD11b^+^C/EBP*β* ^+^S100A8^+^CD33^+^ MMP9^+^ S100A9^+^ IL-4R*α* ^+^.	Fibrocystic, human-GM-CSF, and human-G-CSF.	Tregs, IDO	[88]

40	Mouse	GVHD	CD115^+^Ly-6C^+^ and CD115^+^Ly-6C^−^ cells	Embryonic stem (ES) cells, bone marrow hematopoietic stem (HS) cells	Tregs, IL-10, iNOs	[89]

41	Human		CD14^+^CD33^+^CD34^+^	Peripheral blood mononuclear cells (PBMCs), prostaglandin E2 (PGE2), GM-CSF/IL-4	IDO, IL-4R*α*, NOS2, IL-10	[90]

**Table 2:** Aggressive MDSCs in EAE.

	Species	Diseases or models	Cell surface phenotype	Mechanism of suppression	Reference
1	Mouse	EAE	CD11b^+^ CD62L Ly-6C^hi^	Maturation into functional DCs and/or inflammatory macrophages	[50]

2	Mouse	EAE	CD11b^+^Gr1^+^	Th17, IL-1	[52]

3	Mouse	EAE	CD11b^+^Ly-6C^hi^Ly-6G^−/low^	(i) Induction of MDSC apoptosis; (ii) polarization of MDSCs to mature subsets of myeloid cells (dendritic cells/macrophages/neutrophils); and (iii) altering their immunosuppressor phenotype	[56]
